# Epidemiology of non-traumatic spinal cord injury in Uganda: a single center, prospective study with MRI evaluation

**DOI:** 10.1186/s12883-019-1236-3

**Published:** 2019-01-15

**Authors:** Abdu K. Musubire, David B. Meya, Elly T. Katabira, Ana Claire L. Meyer, Paul R. Bohjanen, David R. Boulware, Frank Minja

**Affiliations:** 1 Infectious Diseases Institute, College of Health Sciences, Makerere University, P.O. Box 22418, Kampala, Uganda; 2 Department of Medicine, School of Medicine, College of Health Sciences, Makerere University and Mulago Hospital Kampala, Kampala, Uganda; 3 Department of Neurology, Yale School of Medicine, Yale University, New Haven, CT USA; 4 Division of Infectious Diseases & International Medicine, Department of Medicine, University of Minnesota, Minneapolis, MN USA; 5 Department of Radiology and Biomedical Imaging ,Yale School of Medicine, Yale University, New Haven, CT USA

**Keywords:** Myelopathy, Non-traumatic spinal cord injury, Sub-Saharan Africa, Mortality, MRI, Uganda

## Abstract

**Background:**

A few reliable national data concerning the etiology of non-traumatic spinal cord injury (SCI) in sub-Sahara Africa exists, mainly because of the limitations of diagnostic imaging. These are both expensive and mostly unavailable in several resource-limited settings. Only a few studies have employed the magnetic resonance imaging (MRI) in documenting non-traumatic SCI and most of these studies are from South Africa. We sought to describe the clinical presentation, MRI radiological patterns, and one-year survival among subjects with non-traumatic SCI in Uganda.

**Methods:**

We enrolled a prospective cohort of 103 participants with non-traumatic SCI at Mulago National Referral Hospital Kampala, Uganda in 2013–2015. Participants received standard of care management, with surgical intervention as needed, with one-year follow up. Data were analyzed using Descriptive statistics.

**Results:**

In 103 participants with non-traumatic SCI, the median (IQR) age was 37 (18, 85) years and 25% of the participants were HIV-infected. Paraplegia/paraparesis was the most common clinical presentation in 70% (*n* = 72). Severe disease was present in 82% (*n* = 85) as per American Spinal Injury Association (ASIA) scale A-C. On MRI, 50% had extradural lesions. However, bone lesions accounted for only 75% of all the extradural lesions. More than 60% of the patients had lesions that could only be diagnosed on MRI. Deaths occurred in 42% (*n* = 44) of participants, with the highest mortality among those with extradural lesions (60%).

**Conclusion:**

The mortality following non-traumatic spinal cord injuries in Uganda is high. We demonstrated an equal distribution between extradural and intradural lesions, which differs from the historical predominance of extradural lesions. Increased utilization of MRI particularly among young age groups is needed to make a diagnosis.

**Electronic supplementary material:**

The online version of this article (10.1186/s12883-019-1236-3) contains supplementary material, which is available to authorized users.

## Background

The estimated global spinal cord injury (SCI) incidence is 40 to 80 new cases per million population per year [1]. These injuries occur more in Africa than elsewhere [2]. Non-traumatic SCI is more common in the older age groups, and hence with increased life spans, the incidence of non-traumatic SCI will increase and overtake that of traumatic SCI [3]. The etiology of non-traumatic SCI is region dependent. Most etiologies found in temperate regions also occur in the tropics, however, infectious and nutritional disorders occur with higher prevalence in tropical regions [4]. HIV infection has been found to influence the causes of non-traumatic SCI with a bias towards infectious causes [5].

The etiological diagnosis of non-traumatic SCI is usually established by collating information obtained from magnetic resonance imaging (MRI) scans, Cerebrospinal Fluid (CSF) analyses, blood studies, Chest X-Ray findings, non-neurological illnesses and responses to treatments [5]. While the computed tomography (CT) will define the vertebral bone lesions, the MRI will provide more spinal cord and soft tissue details. The classification of lesions based on MRI can be compressive verses non-compressive lesions [6]. Alternatively, the lesions may be classified as extradural or intradural lesions basing on the location of the lesion in relation to the dura. Intradural lesions are further classified as intramedullary versus extramedullary lesions basing on the location of the lesion in relation to the spinal cord [7]. Vascular injuries can be evaluated using CT or MR angiography [6] While CT and X-ray are limited to diagnose bony compressive lesions or extradural compressive lesions, MRI is useful in the diagnosis of all types of lesions. Few reliable national data concerning the etiology of non-traumatic SCI in sub-Sahara Africa exists, mainly because of the limitations of diagnostic imaging [1, 2, 8]. These are both expensive and mostly unavailable in several resource-limited settings. Only a few studies have employed the MRI in documenting non-traumatic SCI and most of these studies are from South Africa [5, 9–11]. We sought to describe the clinical presentation, MRI radiological patterns, and one-year survival among subjects with non-traumatic SCI in Uganda.

## Methods

The study site was Mulago National Referral Hospital in Kampala, Uganda between 2013 and 2015. This was a prospective cohort with consecutive sampling. Patients with non-traumatic SCI presented with disorders of the spinal cord resulting in a motor (paraplegia or quadriplegia with upper motor neuron signs or features consistent with spinal shock), sensory (sensory level for pinprick and light touch and/or loss of proprioception and vibration) and autonomic dysfunction (impaired sphincter control). Participants were eligible if they were > 18 years of age, had a non-traumatic SCI and provided informed consent.

Patients with suspected spinal cord injury were identified during their admission on a medical ward at Mulago Hospital. The ward team on identifying the eligible patients invited the study team. The eligible patients were screened, consented and enrolled by a research assistant on the study. Data were collected on a paper questionnaire completed by a pen which was eventually entered into the datafax electronic system. We collected demographic information and the clinical duration of illness from onset of symptoms to hospital presentation. The extent of the injury was defined by the American Spinal Injury Association (ASIA) impairment scale. The radiological imaging was on 16 channel 1.5 T MRI (Philips Achieva 1.5 T, Phillips Healthcare, Netherlands). Imaging was performed with all the patients lying in a supine position using a sense spine coil. Full spinal survey was done by a sagittal T1 weighted sequence (TR/TE 28/4 ms,10 mm thickness, flip angle 45^o^,BW 137 Hz/px; with the following specific protocols: Sagittal spine survey: matrix 288 × 284/288 × 384;DFOV 45 cm × 45 cm/45 × 87 cm, Coronal spine survey: matrix 192 × 256; DFOV 30X30cm/36x57cm. MobiView program was used to stack the images in sagittal and coronal sections. This was followed by a sagittal T1 weighted sequence (TR/TE-400/12 ms; DFOV: 36 × 69 cm; Slice gap - 0.4; Slice thickness - 4 mm; Matrix 280 × 396; Flip angle - 90°; DFOV was 36X36cm) and axial T1-weighted sequence in the cervical areas T1W_TFE_3D (TR/ TE 9/4 ms; slice thickness 3 mm; Flip angle:25^O^; BW 153 Hz/px; DFOV 15X15cm; matrix 188 × 150). We also did sagittal T2 weighted sequence T2W_DRIVE (TR/TE-2659/120 ms; DFOV 36X68cm/36x36cm; Flip angle - 90°; Matrix 291 × 380; Slice thickness 4 mm; and BW 363 Hz/px) and axial T2-weighted sequence in the lumbar region(T2W_DRIVE TR/ TE 3696/120 ms; slice thickness 4 mm; Flip angle:90^O^; BW 431 Hz/px; DFOV 20X20cm; matrix 308 × 212). Short tau inversion recovery (STIR) images were acquired for the different regions of the spine (TR/TE-3500/80 ms; TI 165; DFOV: 36 × 69 cm/36 × 36 cm; Slice thickness 4 mm; flip angle 90^O^; BW 306 Hz/px; matrix 288 × 364). Sagittal whole spine imaging was with gadolinium was done in T1 weighted sequence T1W_TSE + GADO (TR/ TE 400/12 ms; slice thickness 4 mm; Flip angle:90^O^; BW 554 Hz/px; DFOV 36X69cm/36x36cm) and axial Cervical axial scans T1W_TFE_3D + GADO (TR/ TE 9/4 ms; slice thickness 3 mm; Flip angle:25^O^;BW 153 Hz/px; DFOV 15X15cm;matrix 188 × 150).

Variables of interest included the location of the lesion in the longitudinal section (e.g. cervical, thoracic, lumbar, or multilevel) and the number of vertebrae involved. General classifications were according to the lesion location and whether intradural or extradural. We further categorized extradural lesions as either bone or non-bone lesions. We classified intradural lesions as intramedullary or extramedullary. This lesion categorization is presented in Additional file 1: Appendix Table S3. Orthopedic surgeons managed participants with extradural bony lesions, and neurosurgeons managed participants with other compressive lesions. Blood draws for the standard of care tests included HIV enzyme-linked immunosorbent assay (ELISA).

For clinical outcomes, we assessed the one-year mortality and mobility outcomes through quarterly visits and via phone calls for those participants that missed the clinical visits. The attending neurologist’s documented diagnosis was considered as the final diagnosis for this study.

The Makerere University Research and Ethics Committee and the Uganda National Council for Science and Technology provided ethical approval for the study.

## Results

**Fig. 1:**
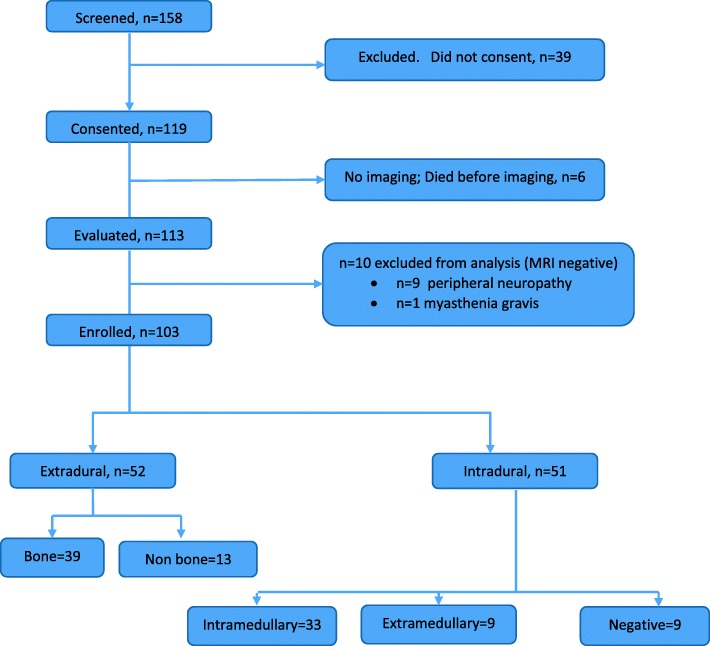
Study Flow Diagram. Patients with no lesions on MRI with strong clinical suspicion for non-traumatic spinal cord injury were categorized under Intramedullary lesions

We screened 158 patients with suspicion for non-traumatic SCI on a medical ward. Of these, 119 provided informed consent and 113 received MRI. Six died before imaging was obtained. Among the 113, 10 patients had alternative diagnosis: peripheral neuropathy (*n* = 9) or myasthenia gravis (*n* = 1) and were excluded. In total, 103 patients with non-traumatic SCI were prospectively enrolled in the study (Fig. 1). The complete dataset is attached as a Additional file 2 in excel format.

Demographics of participants presenting with non-traumatic SCI are presented in Table 1. Men encompassed 52% of participants. The median (IQR) age of 37(18–84) years’ participants with intradural lesions presenting at a young age than those with extradural lesions. One quarter 25% of the participants were HIV-infected with a higher representation among participants in the intradural group. Participants presented late to the hospital with 50% (51/103) reporting symptoms for a duration of more than one month.

**Table 1:** Clinical Presentation and Radiological Presentation and Mortality Outcomes of Patients with Non-Traumatic Spinal Cord Injury in Uganda

Characteristic	Intradural	Extradural	Total	*P*-value
N (%)	51 (50)	52 (50)	103	
Men	22 (41)	32 (59)	54 (52%)	0.24
Age in year, Median (range)	35 (18–84)	44 (18–82)	37 (18–84)	
Age mean ± SD	36 ± 14	44 ± 19	40 ± 17	0.016
Duration of illness in days (mean, +SD)	175 ± 412	163 ± 319	223 ± 644	0.66
Duration of illness in days (median, Range)	28 (1–5569)	38 (2–1616)	31 (1–5569)	
Duration of illness in less than 30 days	27 (53)	24 (46)	51 (50)	
Duration of illness, weeks				
● Acute ≤1 day	3 (6)	0 (0)	3 (3)	
● Subacute ≤1 week	5 (10)	2 (4)	7 (7)	
● Prolonged > 1 week ≤1 month	19 (37)	22 (42)	41 (40)	
● Chronic > 1 month	24 (47)	28 (54)	52 (50)	
Level of the neurologic lesion				0.99
● Quadriplegia / Quadriparesis	15 (29)	16 (31)	31 (30)	
● Paraplegia / Paraparesis	36 (71)	36 (69)	72 (70)	
HIV-infected	17 (33)	9 (17)	26 (25)	0.18
The severity of Injury by ASIA scale				
● A-N	22 (43)	18 (35)	40 (39)	0.98
● Β-C N	21 (41)	24 (47)	45 (44)	
● D N	8 (16)	10 (19)	18 (17)	
Outcomes (after one year)				
● Death	13 (25)	29 (56)	42 (41)	0.040
● Unknown	7 (14)	5 (10)	12 (12)	

**#**Values are n (percentage)

Most participants (70%) had paraplegia/paraparesis at presentation. The location of the lesions was mostly in the thoracic region 52%, Cervical 24%, upper lumber 6%, and 18% multi-level. Among the multi-level lesions, 11% involved all levels - cervical, thoracic and lumbar, 5% thoracic and lumber and 2% cervical thoracic. Approximately 40% presented with severe disease consisting of complete lesions defined as deficits in all motor, sensory and autonomic dysfunction and 82% with ASIA scale A, B, and C.

Extradural lesions were present on MRI in 50% (52/103) of participants. Of these, bone lesions accounted for 75% (39/52) of all the extradural lesions. Thus 39/103 (38%) of patients with bone lesions could be diagnosed using less costly investigations including radiographs or computed tomography (CT).

**Fig. 2:**
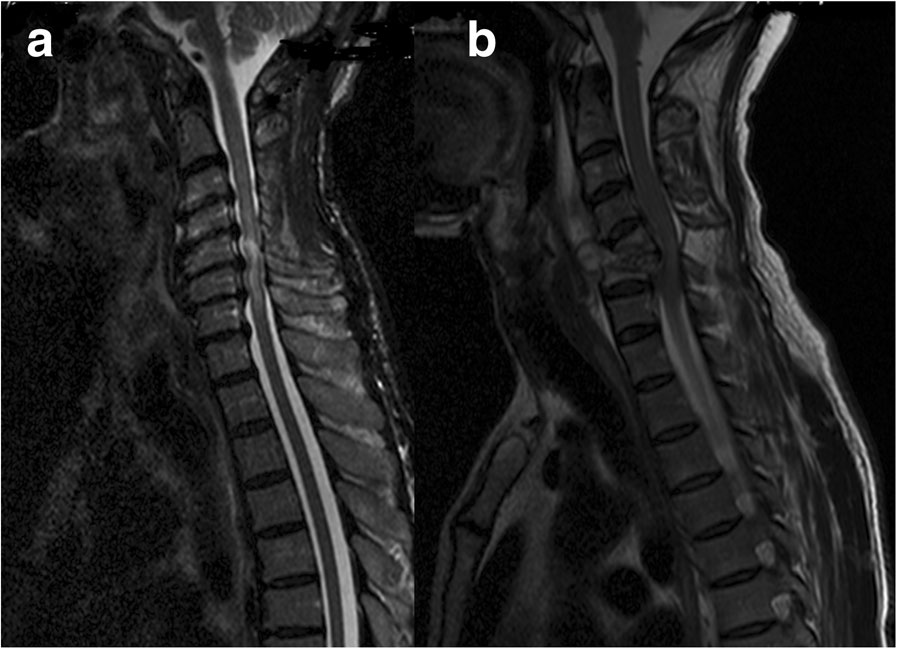
Extradural bone lesions with the diagnosis of degenerative disease and TB infection. **a** Cervical spondylosis diagnosed in a 60–64 year-old participant with quadriparesis for > 2 months. T2 Hyperintensity at C2 and C3 with multiple posterior disc prolapses at C3-C6. The patient died at home 2 months later having refused orthopedic surgery. **b** 45–49 year-old participant presented with progressive quadriparesis for over 3 months with a C4 and C5 vertebra bone destructive lesion who underwent surgery with a biopsy that confirmed acid-fast bacilli consistent with Potts’ disease

Among extradural lesions, the causes were diverse. The following diagnoses were made according to the MRI classification; extradural bony lesions (*n* = 39). Infections predominated by Potts’ disease in 12/103 (12%), suspected tumors (primary and metastases) 18/103 (17%), spondylosis 8/103 (8%) and congenital malformation 1/103 (1%). In the extradural non-bony lesions (*n* = 13), we found suspected tumors in 12/103 (12%) and infection of epidural abscess in 1/103 (1%). (Fig. 2a and b).

**Fig. 3:**
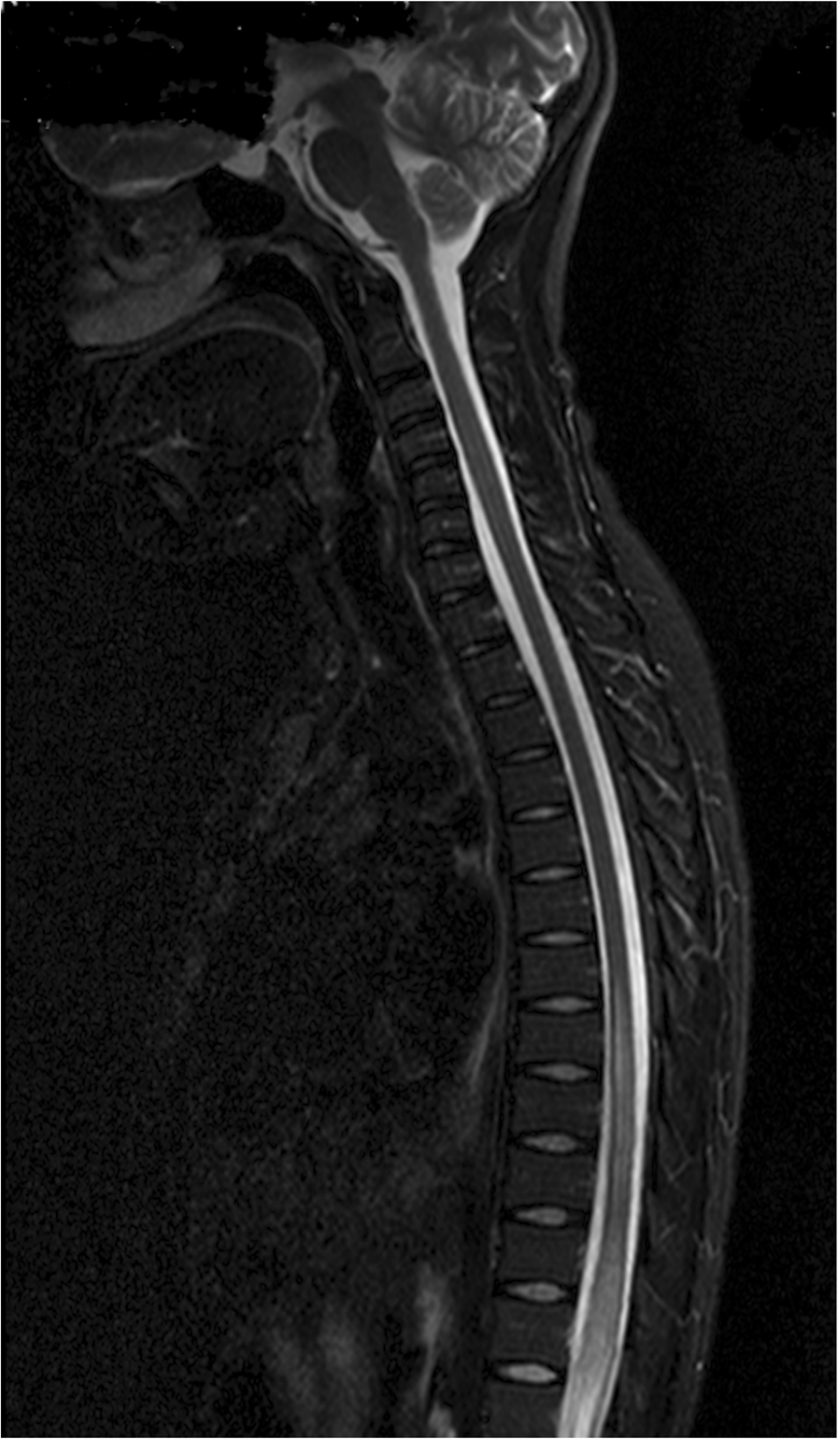
Intradural Lesions with a diagnosis of Transverse Myelitis. T2 hyperintensity lesion T8-T12 in a 20–24 year-old participant who presented with progressive paraplegia over 3 weeks. A diagnosis of transverse myelitis was made and the patient improved on steroids

Intramedullary lesions were diagnosed in 42 participants. Among intramedullary lesions, the principal diagnoses were transverse myelitis in 23% (*n* = 24), (Fig. 3) malignancy in 10% (*n* = 10), ischemia (n = 1), and unknown (*n* = 7) with negative MRI. The intradural extramedullary (*n* = 9), was predominated arachnoiditis in 8% (*n* = 8) due to suspected TB, schistosomiasis, or neurosyphilis. An additional intradural extramedullary lesion was caused by a tumor (*n* = 1).

We assessed participant outcomes through one year of follow up. Mortality occurred in 42% (44/103) of participants, and 12% (*n* = 12) of participants were lost to follow up. Most early deaths occurred in the first 12 weeks (Additional file 3: Figure S1).

Participants with extradural lesions experienced 56% (29/52) mortality compared to 25% (13/51) mortality with intramedullary lesions (*P* = 0.006). Death was two times more likely in the extradural group than the intradural group, increased with increasing age and 5 times higher in participants with a poor initial ASIA scale (Table 2).

**Table 2:** Factors Associated with Mortality

Category	Hazard Ratio	95% CI	*P* value	Adjusted Hazard Ratio	95% CI	*P* value
MRI Extradural Lesion	2.6	1.3–5.4	0.008	2.2	1.2–3.9	0.008
Age (per 10-year increase	1.2	1.0–1.4	0.007	1.2	1.0–1.3	0.019
Sex (Male)	1.1	0.6–1.9	0.6	1.2	0.7–2.0	0.56
HIV-infected	0.9	0.45–1.6	0.43	1.1	0.5–2.2	0.69
ASIA scale	3.6	1.4–8.9	0.007	4.6	1.6–13.1	0.003

Among the survivors, *N* = 47(46%), five (5%) were bedridden, seven (7%) wheelchair-bound, six (6%) able to walk with the help of canes and 29(28%) walk unsupported.

Obtaining definitive diagnoses was a challenge in this study. Five biopsies performed on the extradural lesions that confirmed Potts’ disease in four participants and unclassified bone tumor in one. One biopsy for intradural tumor demonstrated a lymphoma in an HIV-infected participant and another biopsy for intramedullary tumor demonstrated a germ cell tumor. Thirty participants had CSF analyzed, with 5% of the samples having pleocytosis. Given the following diagnostic challenges, low lumbar puncture acceptance rates, the limited CSF diagnostic analyses, and the limited number of biopsies done, most of the diagnoses depended on the clinical presentation, imaging findings, and response to treatment.

## Discussion

To our knowledge, this is the largest longitudinal cohort of MRI evaluated non-traumatic SCI patients in sub-Saharan Africa. We had a 12% loss to follow up through one year. We have found an almost equal distribution of extradural to intradural lesions on MRI and a high one-year mortality of 40%.

We found that non-traumatic SCI mainly affects the young productive age group of 30–40 year-olds, similar to many other African studies [5, 11]. The male to female ratio was almost 1: 1, comparable to other studies in the Sub Sahara region [5, 10]. The HIV prevalence of 25% among patients with non-traumatic SCI was three times the national HIV seroprevalence of 7–8% in Uganda [12]. The HIV prevalence rates among our participants with non-traumatic SCI is more than in Nigeria(14%) [10] and Ethiopia(16.9%) [13], but less than South Africa(50%) [5]. HIV may be a factor in increasing the prevalence of non-traumatic SCI. Overall, 50% presented at or after one month, like in other African studies [2, 10]. The reasons for the late presentation that have been reported in other studies include insidious onset and slow progress of the disease [13, 14], belief that the disease is a result of sorcery hence seeking traditional remedies before coming to the hospital, financial and transportation difficulties [10, 15]. Participants also presented with severe disease as manifested by complete lesions and poor ASIA scales (ABC).

MRI is the gold standard imaging technique for spinal cord pathology; however, this modality of investigation is still largely unavailable in most centers in sub-Saharan Africa. Approximately 50% of participants had extradural lesions on MRI and bone lesions accounted for 76% (39/52) of extradural lesions. This would give us a proportion of 38% (39/103) participants with lesions that can be diagnosed by bone-based investigations like X-rays and CTs leaving 62% undiagnosed. Historically, most of the spinal cord pathology in Africa with a diagnosis has been compressive bone lesions with about 60% from Potts’ disease and tumors [1, 8]. Our results show a change in the trend with more non-bone extradural lesions and intradural lesions. This may be because a number of these studies were from neurosurgical wards [11, 16] compared to this study conducted on a medical ward. The other reason could be referral bias, as this study did not have access to participants with possible bony lesions admitted directly to the orthopedic spine wards. They may also be change in the trend of the presentation with more participants presenting with intradural lesions. Our results highlight the increasing significance of MRI among patients with non-traumatic SCI admitted on in the medical or neurology ward since over 60% of the patients will not benefit from the radiation-based investigations X rays and CT. The differential diagnosis of non-traumatic spinal cord injury is wide [1, 8]. This provides a challenge in choosing the appropriate cheaper investigational modalities. Hence, MRI provides a diagnostic entry point that can guide the use of other investigative modalities for conditions like, infections, demyelination or vascular causes [7, 17]. Prioritization of MRI over CT is mainly in patients with suspected non-bone extradural lesions and intradural lesions which include the younger age groups. Aside from the cost, there is no radiation exposure in MRI.

Among the 51 participants presenting within 4 weeks of symptom onset, 31 of these had either an intramedullary lesion or a negative MRI. Nine of these were HIV-infected. This cutoff of one month is helpful for screening for transverse myelitis [18, 19]. The several negative MRIs, which could have been due to late presentation by the patients as described previously [20, 21].

Mortality occurred in approximately 40% of patients, with about 12% of the patients lost to follow up. The highest mortality was among patients with extradural lesions, 56% mortality. This is higher than what was found in Zimbabwe 18% and in other parts of the world [22]. However, the Zimbabwe study had a high loss to follow up of 34% [22]. A study done in Israel of 1085 patients with non-traumatic SCI admitted between 1962 and 2000 reported the cumulative mortality was 0.6% after the first year, 6% at 5 years and 16% after 10 years [1, 23]. The high mortality rate could be because of the delay in seeking care, the severity of presentation and lack of rehabilitation facilities. Most of the early deaths occurred in the first 12 weeks and was associated with extradural lesions on MRI categorization, increasing age and poor ASIA scale. However, survivors had a good mobility state with the majority able to walk in one year.

The limitations of this study include the small sample size of only 103 patients, a single center study and the wide differential diagnosis that makes the generalization of the results difficult to any etiological agent. We also had several patients unwilling to report for the physical exam at termination but agreed to a telephone interview limiting the functional outcomes to self-reported mobility outcomes.

## Conclusion

To our knowledge, this is the largest MRI series of non-traumatic SCI is sub-Saharan Africa. We have demonstrated different characteristics of non-traumatic SCI on a medical ward compared to previous studies, with our finding of fewer extradural bone lesions and more intradural lesions. This change calls for more utilization of MRI studies, especially in young patients. The high mortality rate in patients with non-traumatic SCI calls for a need to improve treatments and outcomes in this population.

## Additional files

Additional file 1: **Appendix Table S1**: Categorization of lesions by MRI anatomical location (DOCX 12 kb)

Additional file 2: A complete dataset in excel has been added as a separate file. (XLSX 31 kb)

Additional file 3: **Figure S1.** Survival Time from Diagnosis of Non-traumatic Spinal Cord Lesion by MRI Classification. (DOCX 26 kb)

## Abbreviations

ASIA: American Spinal Injury Association
BW: Bandwidth
cm: Centimeter
CSF: Cerebrospinal Fluid (CSF)
CT: Computed tomography (CT)
DFOV: Display Field-of-View
ELISA: Enzyme-linked immunosorbent assay
HIV: Human immunodeficiency virus
Hz: Hertz
IQR: Interquartile range
MRI: Magnetic resonance imaging
Px: Pixel
SCI: Spinal cord injury
SD: Standard deviation
STIR: Short tau inversion recovery
TFE: Turbo Field Echo
TR: repetition time and TE echo time
TSE: Turbo Spin Echo
