# Supplementary material for: Epidemiology of non-traumatic spinal cord injury in Uganda: a single center, prospective study with MRI evaluation
Source: BMC Neurol. 2019 Jan 15;19:10. doi: 10.1186/s12883-019-1236-3 (PMC6332574; doi:10.1186/s12883-019-1236-3)
Supplement: Supplementary file 1 — Appendix Table S1: Categorization of lesions by MRI anatomical location (DOCX 12 kb) [file 12883_2019_1236_MOESM1_ESM.docx]

| Extradural | Bony | In the extramedullary group, most of the patients were suspected to have Potts disease, tumors, and degenerative bone disease. |
| --- | --- | --- |
|  | Non-bony | Abscess, infections, tumors |
| Intradural | Extramedullary | In the intradural group most of it was classified under arachdonitis |
|  | Intramedullary | Patients in this category included those with observable intramedullary lesions and a few with negative MRI but clinical presentation suggestive of Spinal cord injury  The commonest diagnoses were transverse myelitis and tumors |
|  |  |  |
