# Supplementary figures and images for: Epidemiology of non-traumatic spinal cord injury in Uganda: a single center, prospective study with MRI evaluation
Source: BMC Neurol. 2019 Jan 15;19:10. doi: 10.1186/s12883-019-1236-3 (PMC6332574; doi:10.1186/s12883-019-1236-3)

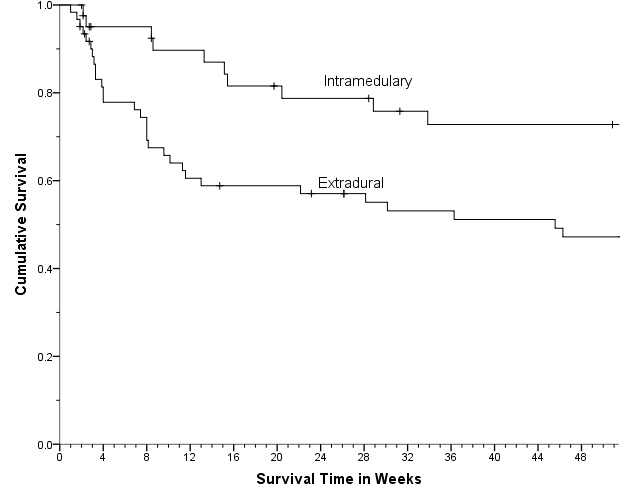

Supplement: Supplementary file 3 — Figure S1. Survival Time from Diagnosis of Non-traumatic Spinal Cord Lesion by MRI Classification. (DOCX 26 kb) [file 12883_2019_1236_MOESM3_ESM.docx]
